# Effectiveness and postoperative pain level of single-port versus two-port thoracoscopic lobectomy for lung cancer: a retrospective cohort study

**DOI:** 10.1007/s11748-020-01479-z

**Published:** 2020-09-08

**Authors:** Cheng-guang Hu, Kang Zheng, Guan-hua Liu, Zhi-long Li, Yan-li Zhao, Jian-hong Lian, Shi-ping Guo

**Affiliations:** Department of Thoracic Surgery, ShanXi Cancer Hospital, the Affiliated Cancer Hospital of Shanxi Medical University, No. 3 Kaixuan Street, Xinghualing District, Taiyuan, 030013 China

**Keywords:** Lung cancer, Video-assisted thoracic surgery, Single port, Lobectomy

## Abstract

**Objectives:**

Single-port thoracoscopic lobectomy is a new therapeutic technique for patients with lung cancer; however, insufficient data are available regarding its clinical outcomes. We therefore compared the clinical outcomes of single-port and two-port thoracoscopic lobectomies for lung cancer.

**Methods:**

We retrospectively analyzed and compared the data of 204 and 368 patients with lung cancer who underwent single-port or two-port thoracoscopic lobectomy, respectively, between October 2014 and October 2017 at our institution. Patients in both groups underwent 1:1 propensity score matching, and 400 patients (200 patients in each group) were included. Perioperative clinical indicators were analyzed, including operation time, lymph node dissection stations and numbers, incidence of postoperative complications, and pain scores at 24 h, 72 h, and 1 week after surgery.

**Results:**

No perioperative deaths occurred in either group. The operation time, intraoperative blood loss, chest drainage duration, duration of postoperative hospital stay, lymph node dissection station and number, rate of conversion to open surgery, number of ruptured intraoperative pulmonary vessel, and incidence of postoperative complications were not significantly different between the groups (all *P* > 0.05). However, analysis of the 24-h (*P* = 0.005), 72-h (*P* = 0.011), and 1-week (*P* = 0.034) visual analog scale score after surgery revealed that the postoperative pain levels were significantly lower in the single-port than in the two-port group.

**Conclusions:**

Single-port and two-port thoracoscopic lobectomies had similar perioperative outcomes, although the postoperative pain was lower after single-port than two-port thoracoscopic lobectomy. Hence, we concluded that single-port thoracoscopic lobectomy is an effective, minimally invasive, and promising surgical procedure.

## Introduction

Lung cancer is a serious malignant disease, with the highest mortality rate of all the malignant diseases [1]. Resection (most commonly lobectomy) is one of the main treatment modalities for lung cancer. Thoracoscopic lobectomy for lung cancer was first performed in the 1990s [2]; furthermore, it is widely favored by thoracic surgeons and patients, as it involves minimal invasiveness, rapid recovery, and maintenance of cosmetic features. Moreover, as surgical technology and instrument have improved over time, the number of ports (incisions) required for thoracoscopic lobectomy has gradually reduced.

In 2011, Gonzalez et al. [3] first reported the use of single-port thoracoscopic lobectomy; this method has subsequently been adopted by thoracic surgeons in multiple medical institutions owing to its conception of minimal invasiveness and distinct characteristics, primarily the use of the same port for the thoracoscope and the surgical instrument. Several studies [4–6] have compared the single-port and the conventional thoracoscopic lobectomies to verify the superiority of this new surgical approach; however, most of these studies were retrospective, and there was a low degree of matching in the control groups.

In this study, we adopted the targeted, effective propensity score matching statistical method to retrospectively compare the clinical outcomes of 204 and 368 patients who underwent single-port or two-port thoracoscopic lobectomy, respectively, to determine the surgical characteristics, postoperative pain levels, and feasibility of single-port thoracoscopic lobectomy.

## Subjects

This study was approved by the ethics committee of our institution (approval number: 2019036). We retrospectively analyzed all the data of patients with lung cancer who were treated using single-port or two-port thoracoscopic lobectomy by the same group of three surgeons between October 2014 and October 2017. All the participants provided informed consent prior to participation. After being provided with complete information regarding both procedures, the patients were asked whether they wished to undergo single-port (which was still novel at the time of the study) or the well-established two-port thoracoscopic lobectomy. The exclusion criteria of the cases of this study were as follows: 1) performance of wedge resection, segmental resection, pneumonectomy, sleeve lobectomy, bilobectomy, or second lobectomy; 2) presence of metastatic, dual-source, or multi-source lung cancer; 3) administration of radiotherapy, chemotherapy, or targeted therapy (including neoadjuvant therapy) before surgery; and/or 4) tumor diameter > 5 cm, diagnosis of mediastinal lymph node enlargement (> 1 cm) before surgery, or obvious pleural thickening or calcification on preoperative chest computed tomography. Finally, a total of 572 eligible patients, divided into the single- and two-port groups (204 and 368 patients, respectively), were analyzed. Propensity score matching was subsequently performed to distribute the patients into two groups in a 1:1 ratio according to their clinical characteristics including sex, age, smoking history, preoperative complications, and pulmonary function.

## Methods

### Surgical methods

#### Posture and incision

**Fig. 1:**
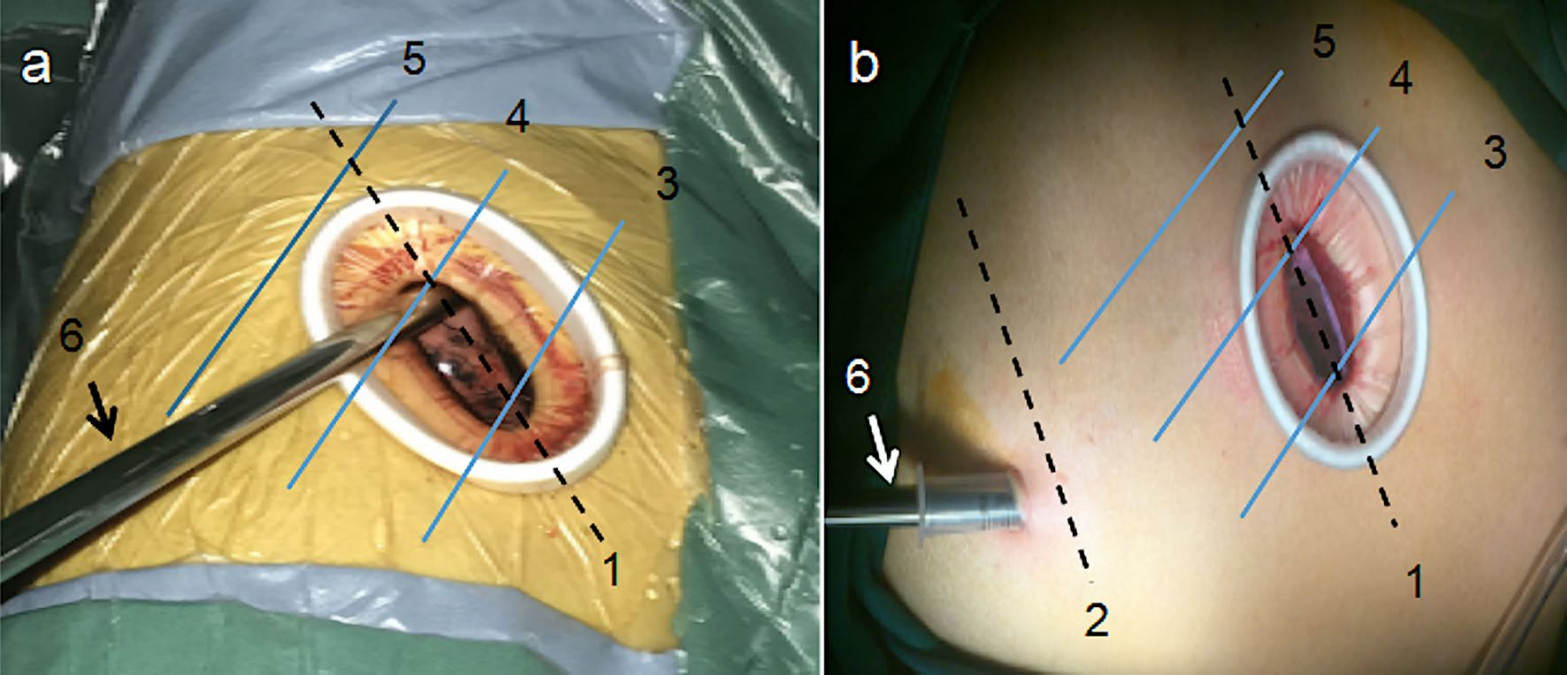
**a** In patients in the single-port group, a single 4–5-cm incision was made at the right fifth intercostal space between the anterior axillary and the midaxillary lines. **b** In patients in the two-port group, an observation port was placed at the right seventh intercostal space along the posterior axillary line, and a 3.5–4.5-cm operation port was placed at the fifth intercostal space between the anterior axillary and the midaxillary lines. 1, the fifth intercostal space; 2, the seventh intercostal space; 3, the anterior axillary line; 4, the midaxillary line; 5, the posterior axillary line; 6, the thoracoscope

All the patients underwent double-lumen endotracheal intubation under general anesthesia while being in the lateral position on the contralateral side. During surgery, the operating bed was flat or folded, and a 30-degree, 10-mm Olympus high-definition lens (Olympus Corporation, Tokyo, Japan) was used. For the two-port group, a 2-cm incision was made at the seventh intercostal space along the posterior axillary line as an observation port, where the trocar was placed. Subsequently, another 3.5–4.5-cm incision was made at the fifth intercostal space between the anterior axillary and the midaxillary lines (Fig. 1). For the single-port group, a 4–5-cm incision was made at the fifth intercostal space between the anterior axillary and the midaxillary lines. This was the sole incision site for the thoracoscope and the surgical instruments. The operation ports for patients in both groups were consistently placed at the fifth intercostal space, regardless of the position of the resected lung lobe, and an elastic incision protector was placed into this operation port (Fig. 1). After resection completion, the pulmonary lobe specimen was placed in a laparoscopic bag and taken out of the body. After surgery, a 26-French thoracic tube was inserted into the incision for drainage in the single-port group, or into the observation port in the two-port group.

#### Pulmonary lobectomy

Patients in both groups underwent anatomic lobectomy. For peripheral lesions without pathology, wedge resection was performed initially. When these lesions were confirmed as lung cancer on frozen-section pathological evaluation, pulmonary lobectomy was performed. In both groups, the tissues were separated or cut with an electrocautery device or ultrasonic knife (Johnson & Johnson, New Brunswick, NJ, USA). The pulmonary vessels, bronchi, and interlobar fissures were clipped with an Endo-GIA stapler (Medtronic, Inc., Minneapolis, MN, USA), while the small vessels were clipped with a HemoLock (Johnson & Johnson, New Brunswick, NJ, USA), or manually knotted with a knot pusher.

#### Systematic lymph node dissection

Systematic lymph node dissection, including that of the hilar and mediastinal nodes, was performed during surgery. During right lobectomy, mediastinal lymph node dissection was performed to remove the following lymph node stations: 2, 3, 4R, 7, 8, and 9; and during the left lobectomy, 4L, 5, 6, 7, 8, and 9 were dissected in both groups.

#### Pain scoring

After surgery, to administer the analgesics, an analgesic pump was connected to the veins and filled with 150 mL of normal saline with 100 μg fentanyl, 20 mg dezocine, and 200 μg dexmedetomidine during the 24 h after surgery according to the body weight (kg). Other analgesic methods such as intercostal nerve block or epidural analgesia were not used. After the removal of the analgesic pump, if the patient still had obvious pain, 30–60 mg ketorolac was injected intramuscularly, every 4–6 h, according to the patients’ age and body weight. Pain was assessed using the 10-grade visual analog scale (VAS) with 0 and 10 corresponding to no pain and extreme pain, respectively. Before surgery, the patient received instructions from the same nurse and completed a questionnaire. The VAS evaluation was conducted twice at each of the following time points: 24 h, 72 h, and 1 week after surgery, and the average score was recorded.

### Statistical analysis

Data were analyzed using SPSS Statistics version 21.0 (IBM Corp., Armonk, NY, USA). Categorical data were expressed as the number of cases (*n*); The Kolmogorov–Smirnov method was used to test the normality of measurement data, and median (quartile) was used for the description of measurement data without a normal distribution. Comparison between groups was performed using the Mann–Whitney *U* test. *P* values < 0.05 were considered statistically significant. Logistic regression was used to calculate the propensity score values according to variables, such as age, sex, smoking status, preoperative complications, and lung function. Patients in the single-port group were matched with those having the closest propensity score in the two-port group; those with widely varying propensity scores were excluded.

## Results

The baseline patient characteristics including sex, age, smoking history, preoperative complications, and pulmonary function were not significantly different between the two groups (*P* > 0.05), although the difference in age (*P* = 0.065) and the forced expiratory volume in 1 s (*P* = 0.088) showed a borderline significance. After propensity score matching in a 1:1 ratio, of the 204 patients in the single-port group, four patients were excluded owing to poor lung functions, indicated by a low forced expiratory volume in 1 s and the low degree of matching. Ultimately, 400 patients (200 in each group) were included. The differences in baseline data between the two groups were further reduced. Among the 400 patients, 221 were men and 179 were women; their median age was 66.00 (56.00, 74.00) years (range 35–81 years) (Table 1). After matching, there were no significant differences in the pathological features between the two groups (*P* > 0.05); 264, 77, 20, and 39 patients had adenocarcinoma, squamous cell carcinoma, small cell carcinoma, and other types of lung cancer, respectively. The tumor locations and patients’ pathological stages, which ranged from Ia to IIIa, were not significantly different between the two groups (Table 2).

**Table 1:** Propensity score matching of general clinical indicators

Category	Before matching				After matching			
	Two-port (*n* = 368)	Single-port (*n* = 204)	*Z*/$\chi^{{2}}$	*P* value	Two-port (*n* = 200)	Single-port (*n* = 200)	*Z*/$\chi^{{2}}$	*P* value
Age (years)	65.00 (56.00,72.00) (31–81)	67.00 (55.00, 77.00) (38–80)	− 1.846^a^	0.065	66.00 (56.00, 72.00) (35–81)	67.00 (55.00, 77.00) (38–80)	− 1.123^a^	0.261
Sex (male)	196 (53.26)	116 (56.86)	0.687^b^	0.407	109 (54.50)	112 (56.00)	0.091^b^	0.763
Smoking	178 (48.37)	102 (50.00)	0.140^b^	0.709	96 (48.00)	99 (49.50)	0.090^b^	0.764
Preoperative complications								
Tuberculosis	10 (2.72)	7 (3.43)	0.232^b^	0.630	6 (3.00)	7 (3.50)	0.080^b^	0.778
Diabetes	19 (5.16)	8 (3.92)	0.450^b^	0.502	10 (5.00)	8 (4.00)	0.233^b^	0.630
Asthma	8 (2.17)	6 (2.94)	0.082^b^	0.775	6 (3.00)	5 (2.50)	0.093^b^	0.760
COPD	18 (4.89)	12 (5.88)	0.259^b^	0.611	9 (4.50)	11 (5.50)	0.211^b^	0.646
Heart disease	26 (7.07)	19 (9.31)	0.915^b^	0.339	17 (8.50)	19 (9.50)	0.122^b^	0.727
Pulmonary function								
FEV1 (%)	91.20 (81.50, 103.96)	90.14 (79.95, 103.40)	− 1.706^a^	0.088	91.32 (81.90,105.27)	90.58 (76.08, 103.82)	− 1.528^a^	0.126
DLCO-SB (%)	88.04 (78.94, 98.24)	87.12 (73.38, 97.61)	− 1.487^a^	0.137	87.99 (78.44, 98.24)	87.12 (73.30, 97.78)	− 1.174^a^	0.240

^a^Mann–Whitney *U* test, ^b^Chi-square test. Values are given as *n* (%) or median (Quartile 1, Quartile 3) deviation

*COPD* chronic obstructive pulmonary disease; *FEV1* forced expiratory volume in one second; *DLCO-SB* diffusing capacity of the lung for carbon monoxide-single breath

**Table 2:** Pathological characteristics after propensity score matching

Category	Two-port (*n* = 200)	Single-port (*n* = 200)	*Z*/$\chi^{{2}}$	*P* value
Pathological type				
Squamous carcinoma	37 (18.5)	40 (20.00)	0.145^b^	0.704
Adenocarcinoma	135 (67.50)	129 (64.50)	0.401^b^	0.527
Small cell carcinoma	9 (4.50)	11 (5.50)	0.211^b^	0.646
Other	19 (9.50)	20 (10.00)	0.028^b^	0.866
Average tumor diameter (cm)	3.15 (2.30, 4.00)	3.15 (2.30, 4.00)	− 0.262^a^	0.794
Tumor location				
Right upper	65 (32.50)	61 (30.50)	0.185^b^	0.667
Right lower	43 (21.50)	39 (19.50)	0.245^b^	0.62
Right middle	10 (5.00)	15 (7.50)	1.067^b^	0.302
Left upper	42 (21.00)	47 (23.50)	0.361^b^	0.548
Left lower	40 (20.00)	38 (19.00)	0.064^b^	0.801
Pathological stage				
Ia	41 (20.50)	36 (18.00)	0.402^b^	0.526
Ib	53 (26.50)	61 (30.50)	0.785^b^	0.376
IIa	51 (25.50)	43 (21.50)	0.890^b^	0.345
IIb	3 (16.50)	29 (14.50)	0.305^b^	0.581
IIIa	22 (11.00)	31 (15.50)	1.762^b^	0.184

^a^Mann–Whitney *U* test, ^b^Chi-square test. Values are given as *n* (%) or median (Quartile 1, Quartile 3) deviation

No perioperative deaths occurred, and none of the lung lobe stumps were positive for cancer cells in either group. Comparison between the single- and two-port groups revealed no significant differences in operation time (156.00 [142.00, 174.00] vs. 157.00 [130.25, 173.75]min), intraoperative blood loss (170.00 [120.00, 240.00] vs. 190.00 [122.50, 240.00]mL), chest drainage volume (340.00 [242.50, 470.00] vs. 390.00 [232.50, 490.00]mL), postoperative hospital stay (5.00 [4.00, 6.00] vs. 5.00 [4.00, 7.00] days), number of lymph node dissection stations (4.00[4.00, 5.00] vs. 4.00 [4.00, 5.00]), number of lymph node dissections (12.00 [10.00, 13.00] vs. 11.00 [10.00, 13.00)]), number of conversions to thoracotomy (8 [4.00%] vs. 9 [4.50%]), or number of ruptured intraoperative pulmonary vessel (8 [4.00] vs. 7 [3.50]). According to the Clavien–Dindo classification system, the incidence of complications after surgery (grades I, II, IIIa, and IIIb) was not significantly different between the two groups (Table 3). In terms of postoperative pain, none of the patients required analgesics 72 h and 1 week after surgery in this study. Every patient was administered analgesics at 24 h after surgery, and no analgesics at 72 h and 1 week after surgery. Notably, the VAS scores after 24 h (3.00 [3.00, 4.00] vs. 4.00[3.00, 5.00]), 72 h (2.00 [1.25, 3.00] vs. 3.00 [2.00, 3.00]), and 1 week (1.00 [1.00, 2.00] vs. 1.00 [1.00, 2.00]) were significantly lower in the single-port than in the two-port group (*P* < 0.05; Table 4).

**Table 3:** Surgical outcomes in two groups after propensity score matching

Category	Two-port thoracoscopic lobectomy (*n* = 200)	Single-port thoracoscopic lobectomy (*n* = 200)	*Z*/$\chi^{{2}}$ /Fisher	*P* value
Operation time (min)	157.00 (130.25, 173.75)	156.00 (142.00, 174.00)	− 0.792^a^	0.426
Intraoperative blood loss (mL)	190.00 (122.50, 240.00)	170.00 (120.00, 240.00)	− 1.359^a^	0.174
Chest drainage volume (mL)	390.00 (232.50, 490.00)	340.00 (242.50, 470.00)	− 0.555^a^	0.579
Postoperative hospital stay (days)	5.00 (4.00, 7.00)	5.00 (4.00, 6.00)	− 1.419^a^	0.156
Number of lymph node dissection stations	4.00 (4.00, 5.00)	4.00 (4.00, 5.00)	− 1.668^a^	0.095
Number of lymph node dissections	11.00 (10.00, 13.00)	12.00 (10.00, 13.00)	− 1.438^a^	0.150
Number of conversions to thoracotomy (cases)	9 (4.50)	8 (4.00)	0.061^b^	0.804
Number of intraoperative pulmonary vessel ruptures (> 500 mL) (cases)	7 (3.50)	8 (4.00)	0.069^b^	0.792
Postoperative complications, *n* (%) Clavien–Dindo complications (cases)	33 (16.50)	35 (17.50)	0.071^b^	0.790
Level I				
Incision infection	4 (2.00)	3 (1.50)	0.000^b^	1.000
Level II				
Arrhythmia	10 (5.00)	12 (6.00)	0.192^b^	0.661
Lower respiratory tract infection	7 (3.50)	6 (3.00)	0.080^b^	0.778
Pulmonary air leak (> 5 days)	8 (4.00)	10 (5.00)	0.233^b^	0.630
Level IIIa				
Bronchopleural fistula	1 (0.500)	0	–^c^	1.000
Atelectasis	2 (1.00)	4 (2.00)	0.169^b^	0.681
Level IIIb				
Second surgery owing to bleeding	1 (0.500)	0	–^c^	1.000

^a^Mann–Whitney *U* test; ^b^Chi-square test; ^c^Fisher exact test. Values are given as n (%) or median (Quartile 1, Quartile 3) deviation. – Not applicable

**Table 4:** Postoperative pain scores

Time	Two-port thoracoscopic lobectomy (*n* = 200)	Single-port thoracoscopic lobectomy (*n* = 200)	*Z*	*P* value
24 h after surgery	4.00 (3.00, 5.00)	3.00 (3.00, 4.00)	− 2.795^a^	0.005
72 h after surgery	3.00 (2.00, 3.00)	2.00 (1.25, 3.00)	− 2.550^a^	0.011
1 week after surgery	1.00 (1.00, 2.00)	1.00 (1.00, 2.00)	− 2.123^a^	0.034

^a^Mann–Whitney *U* test. Values are given as median (Quartile 1, Quartile 3) deviations

## Discussion

In this study, single-port thoracoscopic lobectomy, which is a new technique, achieved similar clinical results, but significantly reduced the postoperative pain compared to the two-port technique. Thus, it seems that the single-port thoracoscopic lobectomy is an effective, minimally invasive, and promising surgical procedure for lung cancer.

It is unclear whether single-port thoracoscopic lobectomy has additional advantages and can replace the traditional two- or multi-port techniques. Moreover, the outcomes of these surgeries vary across institutions [4–6]. Most studies that evaluated the outcomes of single-port thoracoscopic lobectomy were retrospective. In addition, the comparison of these techniques is inevitably complex, as the surgeons’ proficiency, surgical skills, and technical expertise may vary significantly among the different medical institutions (or even in the same surgeon over time). To address this issue, all patients in this study underwent surgery performed by the same surgical group (comprised of three surgeons), during the same period, and at the same hospital. The selection of the single- or two-port technique for the patients was not random. After obtaining comprehensive information regarding the techniques, each patient voluntarily opted for single- or two-port thoracoscopic lobectomy; the surgeons did not influence their choice of surgical technique, which was in line with the tenets of the medical ethics. At the start of the study, only a small number of patients opted for single-port thoracoscopic lobectomy. However, as the technology used during single-port thoracoscopic lobectomy improved over time, more patients opted for this procedure. To obtain more comparable patient data, we adopted the most effective propensity score matching statistical method for analysis. Thus, each patient in the single-port group could correspond with patients who have similar data in the control group, thereby making the comparison more effective and credible.

Single-port technique different from tow-port, for single-port thoracoscopic lobectomy, while stapling the bronchi and pulmonary vessels, it was difficult for the stapler to pass through bronchi and pulmonary vessels at certain angles owing to the lack of auxiliary ports that allow for the placement of such surgical staplers, especially at the upper lobe arteries and veins. This was the primary challenge initially faced by the surgeons who were accustomed to performing two- or multi-port lobectomy [7]. From our experience, it is useful to free the vessels to a sufficient length, remove the lymph nodes and connective tissues that may block the stapler, and pull the bronchi and vessels using threads to allow a flexible stapler to pass through.

Two-port thoracoscopic lobectomy should theoretically be faster and more convenient than single-port lobectomy. In fact, during the initial period of performing single-port lobectomy, the operation time was indeed longer in single-port than in two-port lobectomy; however, the operation time decreased markedly as the surgeons became familiar with the single-port technique. Notably, the operation times for the single- and two-port procedures were not significantly different in this study, indicating that single-port lobectomy can be performed just as expeditiously as two-port lobectomy when surgeons have mastered the technique. In addition, there were no significant differences in any other clinical indicators, including intraoperative blood loss, chest drainage volume, and postoperative hospital stay, between the single- and two-port groups. These findings suggest that the single-port thoracoscopic lobectomy is an effective surgical technique.

In the single-port group, the thoracoscope and operating instruments were placed in the same direction, resulting in easier access and dissection of the regional lymph nodes than in the two-port group. However, the rate of lymph node dissection was not significantly different between the two groups in our study, although the single-port group showed a trend toward improved outcomes, especially after considering the number of lymph node dissection stations (Table 3). Similarly, Liu et al. [8] found that the mediastinal lymph node dissection was easier, and more lymph nodes were removed during single-port lobectomy than during the multi-port procedure.

Single-port thoracoscopy is a relatively new technique. Therefore, a learning curve is involved for surgeons who are accustomed with two- or multi-port surgery. As the stapler can only be used in the thoracic cavity from one direction during single-port lobectomy, we recommend that surgeons should use a rotatable stapler and adjust its angle to be as perpendicular as possible to the vessels or bronchi and close to their roots. In our study, the incidence of postoperative complications according to the Clavien–Dindo classification [9] grades I, II, IIIa, and IIIb, such as bronchopleural fistulas, pulmonary air leaks, or lung infections, were not significantly different between the single- and two-port groups. Therefore, the short-term results of single-port thoracoscopic lobectomy were satisfactory.

Regarding postoperative pain, only a small number of studies have shown a superior effectiveness of single-port video-assisted thoracoscopic surgery compared to two- or multi-port thoracoscopic surgery, whereas most of the studies have shown no significant difference between the two groups [4, 5, 10, 11]. Pain after thoracoscopic surgery is mainly caused by surgical trauma, drainage tube insertion, intercostal nerve injury or compression, and pleural injury. Moreover, the pain is affected by many factors, such as age, anxiety, tension, and other emotions, operation time, surgeon’s skill, complications, and postoperative body position. Unlike the methods used in other studies, in this study, propensity score matching was performed to minimize the differences in baseline characteristics between the two groups. Moreover, surgery was performed by the same three surgeons. Additionally, the postoperative pain is a subjective physical feeling that is influenced by psychological and environmental factors. The VAS scores do not provide an objective quantitative measurement. Hence, to reduce the bias in the patient scores, the same nurse provided detailed information and performed the questionnaire regarding the VAS score of each patient before surgery. Instead of obtaining the rating over the phone, the VAS score evaluation was completed twice at each of the following time points: 24 h, 72 h, and 1 week after surgery for each patient, and the average score was recorded, which further improved the accuracy of pain assessment.

In our study, we found that the VAS scores for postoperative pain at 24 h, 72 h, and 1 week after surgery were significantly lower in patients in the single-port group than in the two-port group. A possible reason for this difference is that the observation port in the two-port group was located at the seventh intercostal space along the posterior axillary line where the chest wall muscles are thick and have abundant blood vessels and nerves. As the intercostal space is narrow, pressing or lifting the thoracoscope squeezes the ribs and intercostal nerves considerably, which was likely to be the main cause of postoperative pain.

To date, several novel single-port thoracoscopic lobectomy methods have been reported, including single-port lobectomy with a 2.5-cm incision [12], single-port subxiphoid thoracoscopic lobectomy [13], single-port lobectomy via the neck [14], and transumbilical single-port lobectomy in an animal model [15]. However, the advancement of thoracoscopic techniques depends on the innovation and development of thoracoscopic instruments. Therefore, we expect that the introduction of cutting-edge technology, such as single-port robots, wireless thoracoscopes, naked-eye three-dimensional thoracoscopes, and flexible surgical staplers with additional joints, would improve the single-port thoracoscopic techniques in the future.

In this study, single-port thoracoscopic lobectomy achieved good clinical efficacy and had significant advantages in terms of reduced postoperative pain. Single-port thoracoscopic lobectomy is an effective, minimally invasive, feasible, and promising surgical procedure in the treatment of lung cancer. This study has several limitations. Specifically, it was a retrospective, single-center study. Moreover, it included a small sample size and was performed in a short study period. Additional studies are needed to clarify whether single-port thoracoscopic lobectomy can replace two- or multi-port procedures to become the primary surgical approach for treating lung cancer [16]. We look forward to obtain additional data from a multicenter prospective study with a large sample to further demonstrate the advantages and disadvantages of single-port and two-port thoracoscopic lobectomies in the treatment of lung cancer.

## Conclusion

Single-port and two-port thoracoscopic lobectomies had similar perioperative outcomes; however, the postoperative pain levels were lower after single-port than two-port thoracoscopic lobectomy. Hence, we concluded that single-port thoracoscopic lobectomy is an effective, minimally invasive, and promising surgical procedure.
